# Dispersal in Host–Parasitoid Interactions: Crop Colonization by Pests and Specialist Enemies

**DOI:** 10.3390/insects9040134

**Published:** 2018-10-05

**Authors:** Edward W. Evans

**Affiliations:** Department of Biology, Utah State University, Logan, UT 84322-5305, USA; ted.evans@usu.edu; Tel.: +01-435-797-2552

**Keywords:** biological control, colonization lag, disturbance, ephemeral habitat, habitat fragmentation, natural enemies, parasitism

## Abstract

Interactions of insect pests and their natural enemies increasingly are being considered from a metapopulation perspective, with focus on movements of individuals among habitat patches (e.g., individual crop fields). Biological control may be undercut in short-lived crops as natural enemies lag behind the pests in colonizing newly created habitat. This hypothesis was tested by assessing parasitism of cereal leaf beetle (*Oulema melanopus*) and alfalfa weevil (*Hypera postica*) larvae at varying distances along transects into newly planted fields of small grains and alfalfa in northern Utah. The rate of parasitism of cereal leaf beetles and alfalfa weevils by their host-specific parasitoids (*Tetrastichus julis* (Eulophidae) and *Bathyplectes curculionis* (Ichneumonidae), respectively) was determined for earliest maturing first generation host larvae. Rates of parasitism did not vary significantly with increasing distance into a newly planted field (up to 250–700 m in individual experiments) from the nearest source field from which pest and parasitoid adults may have immigrated. These results indicate strong, rapid dispersal of the parasitoids in pursuing their prey into new habitat. Thus, across the fragmented agricultural landscape of northern Utah, neither the cereal leaf beetle nor the alfalfa weevil initially gained substantial spatial refuge from parasitism by more strongly dispersing than their natural enemies into newly created habitat. Additional studies, including those of colonization of newly planted crops by generalist pests and natural enemies, are called for in assessing these results with a broader perspective.

## 1. Introduction

Study of insect population dynamics across space and time has shifted increasingly in recent decades from a local focus, to a larger, global perspective that stresses population subdivision and exchange among subpopulations collectively comprising metapopulations. This shift can be seen in the conceptual foundations underlying the biological control of insect pests. There has been increasing emphasis on landscape perspective to understand the dynamics of insect predator–prey interactions, including those of parasitoids and their pest hosts [1,2,3,4,5,6,7,8,9,10].

For many years, the theoretical basis for successful biological control was most often posited to lie in enemy–pest interactions that result in low numbers of pests that are well-regulated locally at stable equilibria [11,12]. Indeed, the interaction of the California red scale (*Aonidiella aurantii*, Hemiptera: Diaspididae) and the wasp *Aphytis melinus* (Hymenoptera: Aphelinidae) provides a well-studied, striking example [13]. An absence of convincing evidence for similar population dynamics in most cases, however, has rekindled interest in the early leads of Nicholson [14] and Huffaker [15] in suggesting that pest–enemy interactions may be best understood as persisting regionally (globally) even as they are characterized locally by unstable population fluctuations and frequent extinctions [16,17,18,19]. As pointed out in recent reviews [20,21], this distinctly different perspective has been widely adopted within the biological control community, as the focus has shifted from past preoccupation with local regulation, to present attention more simply on the magnitude and variability of pest suppression among habitat patches (e.g., among agricultural fields) across landscapes.

While a landscape perspective increasingly is being emphasized for biological control generally (i.e., including successes previously attributed to local pest regulation), the perspective has for many years underlain widely-held intuition concerning how success of biological control may often be limited in short-lived crops, especially annual crops. Thus, frequent disturbance in such crops (e.g., harvesting, replanting, and pesticide application) has been hypothesized to continually disrupt local interactions of pests and their natural enemies, substantially compromising pest suppression in individual fields and typically requiring recurrent immigration of natural enemies from elsewhere [7,17,22,23,24,25,26].

At the heart of the landscape perspective is the importance of pest and enemy dispersal among subpopulations (e.g., as associated with individual crop fields) in which the insects’ numbers fluctuate asynchronously, as proposed by Nicholson [14]. Implicit in the view that frequent disturbance in short-lived crops undermines biological control is that natural enemies recolonize more slowly (or otherwise recover less rapidly) following disruptions than do the pests. Nicholson’s [14] conceptualization can be thought of as “hide and seek” [19], wherein pests initially escape their enemies by out-dispersing them to new patches not yet found by the enemies. Such escape could reduce the magnitude of pest suppression by providing a key, temporary spatial refuge [27] even as it acts to promote global stabilization and persistence of the pest–enemy interaction [17,19,28].

A major impediment to assessing this landscape perspective and the theory supporting it lies in our limited empirical understanding of dispersal patterns and capabilities of natural enemies, especially relative to the pests they attack. Generalist natural enemies are increasingly recognized for their contributions to conservation biological control [29,30], but strong interest continues as well in the potential of specialist natural enemies, including those introduced in classical (importation) biological control [20]. Among the various groups of natural enemies, specialist parasitoids in particular may often be weak dispersers [7,31], but a limited data base for these insects’ dispersal under natural field conditions [32,33,34,35] leaves unresolved how widely this hypothesis holds. Of special interest are the relative dispersal capabilities of mobile insect pests and their specialist parasitoids in fragmented agricultural landscapes in which habitat patches are frequently disturbed or destroyed and newly created.

Here, I explore in particular the ability of specialist parasitoids to pursue their pest hosts into newly planted field crops during initial colonization. I focus on dispersal of specialist parasitoids introduced in past years as classical biological control agents for each of two prominent pests of field crops in western North America, the cereal leaf beetle (CLB; *Oulema melanopus* (L.); Coleoptera: Chrysomelidae) and the alfalfa weevil (*Hypera postica* (Gyllenhal)); Coleoptera: Chrysomelidae). I examine in particular whether upon emergence in the spring the pests may initially disperse more strongly than their biocontrol agents in spreading into new habitat, thereby gaining spatial refuge from parasitism in newly planted crops.

## 2. Materials and Methods

### 2.1. Study System

The study was carried out in Cache Valley in northern Utah. Most of the valley floor is occupied by a patchwork of agricultural fields that vary in size between 10 and 40 ha. Individual fields in any given year are planted primarily to small grains (wheat, barley, and oats) or alfalfa, but also less often to a variety of other crops including peas, lentils, radishes, corn, and safflower. In most cases, neighboring fields are contiguous or separated by gravel roads or dirt tracks. For fields of small grains and alfalfa, the nearest neighboring field is often one in which the same crop (small grains or alfalfa) was grown in the previous year and/or is grown also in the current year.

The two host–parasitoid interactions addressed in this study are similar in many ways. Throughout Cache Valley each year, individual fields of small grains and alfalfa are infested to varying degree by CLB and alfalfa weevils, respectively, both of which are Old World pests introduced accidentally and now spread across much of North America [36,37,38,39,40]. Although some adults of the single generation per year of each pest overwinter in the fields in which they matured, most adults leave these fields during the summer to overwinter elsewhere and disperse early in the following spring to small grain or alfalfa fields [41,42]. The adults lay eggs on young grain foliage (CLB) or in young alfalfa stems (alfalfa weevil), with larvae thereafter feeding on the foliage in May and June before dropping from the host plant to pupate in the soil (CLB [43]) or at the base of the host plant (alfalfa weevil [36]). The heavy feeding damage inflicted by larvae of these two pest species triggered classical biological control releases in Utah of the Old World, host-specific, larval endoparasitoids *Tetrastichus julis* (Walker) (Hymenoptera: Eulophidae) [44,45], and *Bathyplectes curculionis* (Thomson) (Hymenoptera: Ichneumonidae) [46]. After killing the host at completion of its pupal cocoon, and then overwintering within the host cocoon in small grains or alfalfa, both species of wasps emerge as adults in the spring to seek out host larvae on host plant foliage [46,47,48]. Although the rate of parasitism can vary considerably among individual fields, both parasitoids parasitize overall a substantial percentage of their hosts each year in Cache Valley, with rates of parasitism often exceeding 50% at any given site and date [41,49,50].

### 2.2. General Design of Field Studies

The field studies presented here focused on the extent to which emerging parasitoid adults in the spring dispersed from their natal fields into newly created habitat (i.e., into fields that were newly planted with small grains or alfalfa) and succeeded in finding their hosts at successively greater distances into the new fields. The percentage of host larvae parasitized at a given distance was used as a measure of colonizing ability of the parasitoids relative to such ability of the pests, adults of which had also dispersed in the same spring into the new fields to lay eggs. Local rates of parasitism in new habitat, as measured here, allow assessment of successful parasitoid colonization in reflecting both the ability to disperse to the new habitat and the ability to find the host within the new habitat. For CLB and *T. julis*, the field study reported here replicated a similar field study reported previously [51], with the goal of testing the robustness of conclusions drawn from the results of that study. The basic design of the field study in small grains was then extended to alfalfa fields in order to assess how host–parasitoid colonization patterns of the alfalfa weevil–*B. curculionis* compared with those of CLB–*T. julis*. No insecticides were applied to study fields either in the year preceding or in the year of field sampling.

The field studies were conducted using transects laid out to include the maximum distance into new fields from the nearest potential source of dispersing adults (i.e., the closest edge of the nearest neighboring field planted to the same crop in the previous year). The potential effects of wind in facilitating pest and parasitoid dispersal were not addressed. It is noteworthy, however, that growers’ planting decisions coincidentally resulted in each of the newly planted fields studied lying west (i.e., typically upwind) from the nearest source field. To test for maximum potential for spatial refuge from parasitism following pest colonization of newly created habitat, these transects were sampled in late spring when the earliest maturing larvae of the host began to appear in their final (fourth) instar on the crop foliage in each study field in each year (the specific dates when sampling occurred (given below) were determined by visiting the study fields repeatedly during the spring as the host larval populations developed). Fourth instars were selected for study, as previously it was determined that the rate of parasitism of CLB larvae in the field rose as the larvae progressed from first to fourth instar, reflecting that they were at risk of parasitism throughout the larval period [51]. These larvae were sampled along the study transects by sweeping, using a net 38 cm in diameter. Sweep samples were taken on calm, clear days between 900 and 1500 h by swinging the net 180° through the upper canopy for each sweep. Upon collection by sweeping, the samples of CLB and alfalfa weevil larvae were taken to the laboratory and frozen, with fourth instars subsequently counted in each set of sweeps and dissected to assess for parasitism.

### 2.3. Field Study in Small Grains

Dispersal of CLB and *T. julis* into a newly planted field of winter wheat was studied in late spring 2016. The field, located near Cove, Utah, was privately farmed. The field had lain fallow in the spring and summer of the previous year (2015), and given the absence of an extended diapause in *T. julis* [51], it was without an overwintering population of the parasitoid. The field was planted with winter wheat in October 2015 and was colonized by CLB and *T. julis* adults in spring 2016. A sampling transect was laid out that extended 400 m into the field from its eastern edge. This eastern edge was bordered by an adjacent field that had been planted with spring wheat in 2015, but was left fallow in 2016. This adjacent field had harbored parasitized CLB larvae in 2015, and provided the nearest potential source of colonist pest and parasitoid females at all sampling stations along the transect in the newly planted wheat field in spring 2016.

On 3 June 2016, when most CLB larvae were second and third instars, and when fourth instars were first appearing on the wheat foliage in the study field, these larvae were sampled by taking sweep samples at each of fourteen distances (at 1, 5, 10, 20, 40, 80, 120, 160, 200, 240, 280, 320, 360, and 400 m) along the transect into the field from its eastern edge. Two sets of ten sweeps each were taken at each distance by sweeping the wheat along sub-transects placed perpendicular to the main transect. The wheat stood 60–80 cm tall and the seed heads were emerging. Sweep samples were frozen and subsequently all CLB fourth instars collected in each set of ten sweeps were dissected to assess for parasitism.

### 2.4. Field Studies in Alfalfa

Dispersal of alfalfa weevils and *B. curculionis* into newly planted fields of alfalfa was studied in late spring 2017 and 2018. In both years, the study fields had been planted with alfalfa the previous summer, having been planted previously in corn for several years. Upon germination in early summer following seeding, the alfalfa in these fields emerged after alfalfa weevil larvae in surrounding established alfalfa fields had completed development earlier in the growing season. Sweep sampling and visual inspections during the summer of the newly establishing alfalfa confirmed that alfalfa weevils and parasitoids were absent in the newly planted fields. Adults of the weevil and parasitoid dispersed into the fields the following spring, and produced the first generation of alfalfa weevil and parasitoid larvae in these newly planted alfalfa fields; these first-generation larvae were sampled along study transects placed into the fields.

In spring 2017 at the Utah Agricultural Experiment Station (UAES) Animal Science Farm, a sampling transect was laid out that extended westward into a newly planted alfalfa field from its eastern edge. This eastern edge was bordered by a single-lane dirt track, to the east of which lay another alfalfa field. This second field had been planted with alfalfa several years previously and was the nearest potential source of weevil and parasitoid colonists for the five sampling stations set at 10, 50, 100, 150, and 250 m along the transect into the field newly planted with alfalfa (the distance of 200 m was not included, as this low-lying part of the field had suffered heavy loss of alfalfa from waterlogged soil early in the spring). On 23 May 2017, four samples of five sweeps each were taken radiating out from each of the five sampling stations. In addition, six samples of five sweeps each were taken 100 m east into the adjacent established alfalfa field across the dirt track. The alfalfa sampled in the two fields was 30–55 cm tall. Sweep samples were frozen and subsequently all alfalfa weevil fourth instars collected in each set of five sweeps were counted. Sixty of these fourth instars collected at each distance along the study transect and in the adjacent field were dissected to determine whether they were parasitized by *B. curculionis*.

In spring 2018 at the UAES Cache Junction Farm, a sampling transect was laid out that extended 700 m into a newly planted alfalfa field from its eastern edge. This eastern edge was bordered by a gravel road, to the east of which lay another alfalfa field. A stand of alfalfa established several years previously grew in this second field, and this established stand served as nearest source of weevil and parasitoid colonists for each sampling station along the transect into the field newly planted with alfalfa. These sampling stations were set at eight distances (10, 100, 200, 300, 400, 500, 600, and 700 m) into the field. On 18 May 2018, six samples of five sweeps each were taken radiating out from each station point along the transect in the newly planted alfalfa field. Also, twelve samples of five sweeps each were taken in the adjacent established alfalfa field, along a 60 m transect headed east and centered at 100 m into this field from the dirt road separating the two fields. Immediately upon transferring the contents of a five-sweep sample to a plastic bag in the field, the sealed bag was examined by eye to record the number of adult *B. curculionis* present (these distinctively marked, active adults were readily observed and counted as they moved about in the bag). The alfalfa sampled in the two fields was 35–55 cm tall. Because alfalfa weevil larvae occurred in very low numbers in 2018 throughout both fields, additional sweeps to obtain more individuals for dissection were taken along sub-transects extending up to 30 m from each station point and perpendicular to the transect in the newly planted alfalfa field, and 10 m south of and parallel to the transect in the second established alfalfa field. Thirty fourth instars collected at each distance along the study transect and in the adjacent field were dissected to determine whether they were parasitized by *B. curculionis*.

### 2.5. Analyses

All statistical analyses were conducted using SAS [52]. Spearman rank correlations were used to test for relationships between distance into newly planted fields and local densities, as assessed by sweep sampling, of fourth instars of CLB (2016), fourth instars of alfalfa weevils (2017 and 2018), and adults of *B. curculionis* (2018). Logistic regression was used to test for relationships between distance into newly planted fields and local rates of parasitism of fourth instars of CLB by *T. julis*, and of alfalfa weevils by *B. curculionis*. Ln-transformed numbers per five sweeps of alfalfa weevil fourth instars were compared between samples from newly planted vs. adjacent established alfalfa in 2017, using analysis of variance (ANOVA). Square-root transformed numbers per five sweeps of alfalfa weevil fourth instars, or *B. curculionis* adults, were compared between samples from newly planted vs. adjacent established alfalfa in 2018, using ANOVA. The percentages of alfalfa weevil fourth instars parasitized by *B. curculionis* in newly planted vs. adjacent established alfalfa were compared using chi-square analysis.

## 3. Results

**Figure 1:**
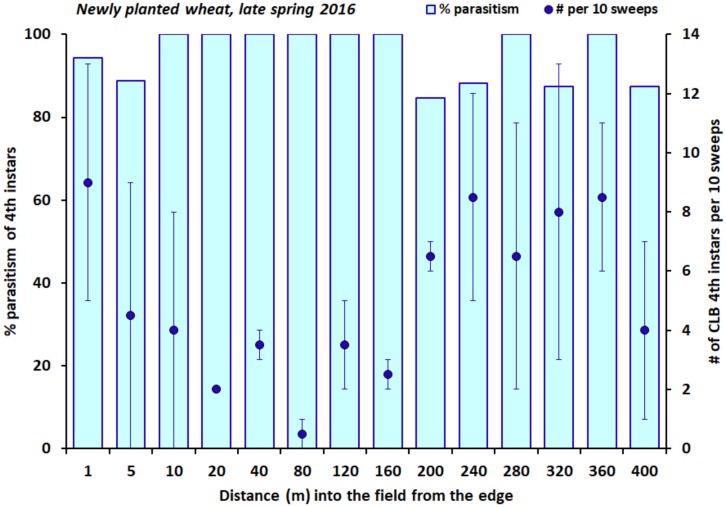
The percentage of fourth instars of the cereal leaf beetle (CLB) that were parasitized by *T. julis*, and the number of CLB fourth instars per ten sweeps (±one SE), at varying distances into a newly planted field of winter wheat in late spring 2016.

Very few CLB larvae occurred in the newly planted winter wheat field sampled in spring 2016. An overall mean of 5.1 ± 0.8 [SE] fourth instars per set of ten sweeps was recorded along the 400 m transect on 3 June 2016. The local density of larvae did not decline significantly with increasing distance into the field from the edge (Figure 1; Spearman rank correlation: *n* = 28, r_s_ = 0.21, *p* = 0.28). Parasitism of these fourth instars by *T. julis* was consistently very high at all distances and did not decline with distance from the field edge (Figure 1; logistic regression: χ^2^ = 0.31, df = 1, *p* = 0.58), nor was it related to local host (CLB fourth instar) density (logistic regression: χ^2^ = 0.66, df = 1, *p* = 0.42). Overall, 94% of fourth instars were parasitized.

**Figure 2:**
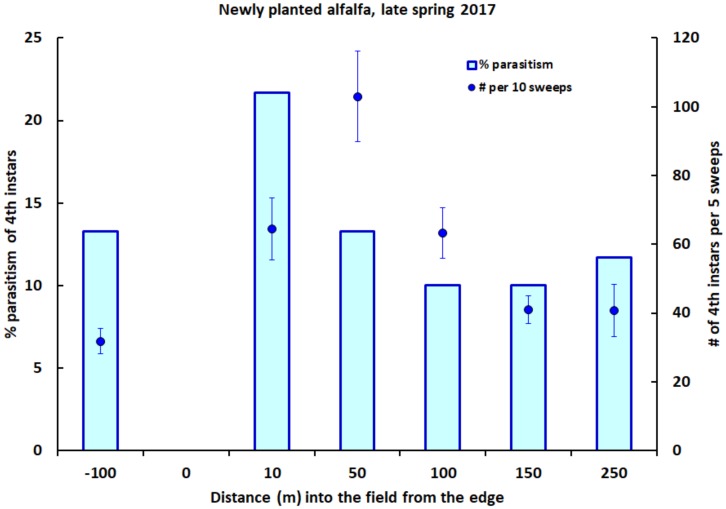
The percentage of fourth instars of the alfalfa weevil that were parasitized by *B. curculionis*, and the number of weevil fourth instars per five sweeps (±one SE), at varying distances into a newly planted field of alfalfa, and at 100 m into an adjacent field of established alfalfa (left end of *x*-axis), in late spring 2017.

Alfalfa weevil larvae were abundant throughout the newly planted alfalfa field sampled in spring 2017. The mean number of fourth instars per five sweeps varied between 40 and 100 larvae at individual distances along the sampling transect (Figure 2). Highest densities occurred within the first 100 m along the transect, with the number of fourth instars per five-sweep sample declining with increasing distance into the field from its edge (Spearman rank correlation: *n* = 20, r_s_ = −0.66, *p* < 0.002). Overall, the mean number of fourth instars per five sweeps was greater in the newly planted alfalfa (62.5 ± 6.3, *n* = 20) than in the neighboring established alfalfa (31.8 ± 3.8, *n* = 6; ANOVA with ln transformation: F_1,24_ = 10.89, *p* = 0.003). Relatively few larvae in either field were parasitized (Figure 2), with the highest local percent parasitism by *B. curculionis* (recorded at 10 m into the new field) reaching only 21.7%. Across all sampling stations along the transect in the newly planted alfalfa, percent parasitism did not differ significantly with increasing distance from the field edge (Figure 2; logistic regression: χ^2^ = 2.00, df = 1, *p* = 0.16). The overall percentage of fourth instars parasitized throughout the newly planted alfalfa was the same as the percentage parasitized in the neighboring established alfalfa (13.3% in both fields).

**Figure 3:**
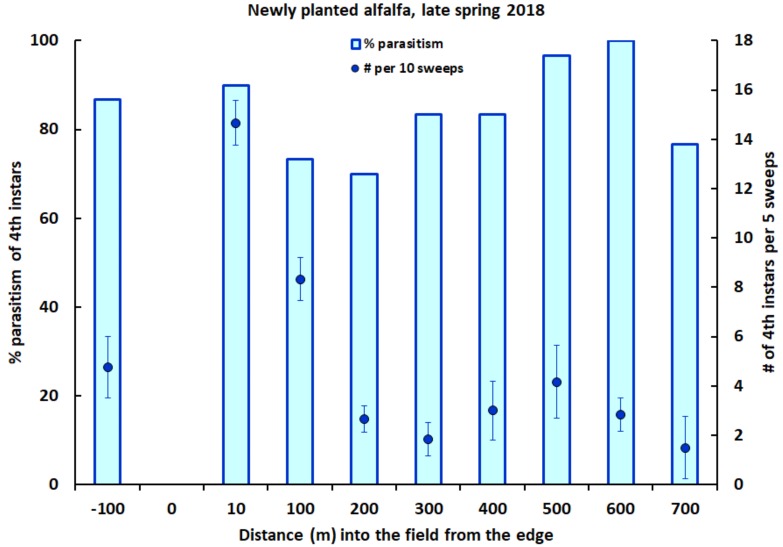
The percentage of fourth instars of the alfalfa weevil that were parasitized by *B. curculionis*, and the number of weevil fourth instars per five sweeps (±one SE), at varying distances into a newly planted field of alfalfa, and at 100 m into an adjacent field of established alfalfa (left end of *x*-axis), in late spring 2018.

Small numbers of alfalfa weevil larvae occurred in the newly planted alfalfa sampled in spring 2018. The mean number of fourth instars varied between 1.5 and 14.7 larvae per five sweeps at individual distances along the sampling transect (Figure 3). As in 2017, the number of fourth instars per five-sweep sample declined with increasing distance from the field’s edge into the newly planted alfalfa, with highest densities of fourth instars occurring within the first 100 m of the transect (Figure 3; Spearman rank correlation: *n* = 48, r_s_ = −0.59, *p* < 0.0001). The mean number of fourth instars per five sweeps was significantly greater than in the neighboring established alfalfa (Figure 3) at up to 100 m along the transect into the newly planted alfalfa field (ANOVA with square-root transformation: F_1,22_ = 13.39, *p* < 0.002), and significantly less overall at 200 m or more (ANOVA with square-root transformation: F_1,46_ = 4.72, *p* = 0.035).

**Figure 4:**
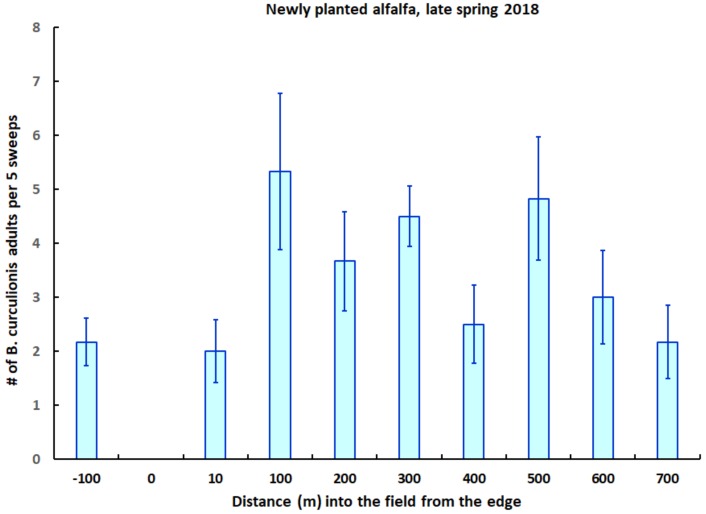
The number of adults of the parasitoid *B. curculionis* (±one SE) at varying distances into a newly planted field of alfalfa, and at 100 m into an adjacent field of established alfalfa (*left end of x-axis*), in late spring 2018.

Adults of *B. curculionis* occurred abundantly in both the newly planted and previously established alfalfa during sampling in 2018, with a mean of 2 to 6 individuals per five sweeps occurring at individual locations (Figure 4). In the newly planted field, the number of *B. curculionis* adults was not related to either the distance from the field edge (Spearman rank correlation: *n* = 48, r_s_ = 0.16, *p* = 0.27) or to local host (weevil fourth instar) densities (Spearman rank correlation: *n* = 48, r_s_ = −0.13, *p* = 0.36). The overall mean number of *B. curculionis* adults per five sweeps was significantly higher in the newly planted alfalfa than in the adjacent established alfalfa (4.0 ± 0.4 vs. 2.2 ± 0.4; ANOVA with square-root transformation: F_1,58_ = 6.00, *p* < 0.02).

Mirroring results for parasitoid adults, the percentage of alfalfa weevil fourth instars that were parasitized by *B. curculionis* in 2018 in the newly planted alfalfa did not differ significantly with distance from the field edge (Figure 3; logistic regression: χ^2^ = 1.98, df = 1, *p* = 0.16) and was not related to the differences in host density among these distances (logistic regression: χ^2^ = 0.24, df = 1, *p* = 0.62). The overall percentage of weevil fourth instars parasitized by *B. curculionis* throughout the newly planted alfalfa (84.2%) was very similar to the percentage parasitized (86.7%) in the neighboring established alfalfa (χ^2^ = 0.13, df = 1, *p* = 0.72).

## 4. Discussion 

Despite the daunting challenges of observing and measuring parasitoid dispersal [32,34,35,53], sustained efforts over many decades have provided numerous insights. Many factors are now well-recognized to influence individual adult parasitoids in their dispersal behavior. These include extrinsic factors such as weather (wind, rain, and ambient temperature [54,55,56,57]) and habitat attributes (e.g., host plant architecture, density, dispersion, and chemical cues [58,59,60]), and intrinsic factors (e.g., physiological status as affected by access to hosts and food [53,61]). Innovative approaches continue to provide new avenues for further progress [62,63,64].

As influenced by these multiple factors, adults of a number of small parasitoid species appear to disperse only up to tens of meters per generation [33,34,55,65,66,67]. Adults of other species, in contrast, disperse hundreds to thousands of meters, up to several kilometers or more [68,69,70,71,72,73]. As with bees [74], dispersal capacity of parasitoids may be positively related in general to body size (e.g., [71]). However, even among studies of closely related parasitoid species of similar size, dispersal capacity can vary markedly. For example, while most field release studies of *Trichogramma* species indicate limited dispersal of tens of meters or less of these very small, egg parasitoids [60,75,76], studies of *T. ostriniae* in corn revealed that individuals dispersed 175–180 m within a week of their release [77,78]. Similarly, among another group of egg parasitoids, the minute fairy flies (Mymaridae), *Anagrus columbi* has very limited dispersal [79], in contrast to *A. delicatus*, which disperses more than 1 km over water to reach isolated habitat [68]. Strong dispersal ability can lead to rapid spread of introduced parasitoids into new environments upon initial release in classical biological control (e.g., [80,81,82]).

In sum, there is a growing, but still substantially restricted, general understanding of dispersal and its implications for population dynamics of host–parasitoid interactions. A case in point concerns the relative dispersal rates of host and parasitoid among habitats and habitat patches [71,72,83,84]. These can reflect differences in the spatial scale at which parasitoids and their hosts respond to the landscape, a topic recognized both as important and as yet little studied [1,4,5,9,85,86,87]. Of particular interest are the relative rates of dispersal of host and parasitoid into newly created habitat [83,88,89] and into existing habitat patches in which previous populations have become extinct [59,71]. Present understanding of the dispersal of prey versus parasitoids and other natural enemies under field conditions remains limited [4,5,9,90,91]. Strong parasitoid dispersal relative to the host could limit host potential for population spread and growth in new habitat [70,92]. Alternatively, relatively weak dispersal could result in the parasitoid lagging behind the host when establishing in newly available habitat (i.e., colonization lag) and thereby failing to prevent host outbreaks. This has often been hypothesized, in general, for insect pests and their natural enemies in short-lived crops subject to frequent disturbance [22,23,24,25,26,29,83,93].

In the present study, the parasitoids dispersed into newly planted crops as strongly, or even more so, than did the host pests. The small eulophid parasitoid *T. julis* (2 mm adult length) readily dispersed hundreds of meters into newly planted wheat upon emerging early in the growing season, as it parasitized almost all of the early maturing CLB larvae within the field. These results build on similar previous findings [51] in revealing the parasitoid’s very strong colonizing ability to disperse deeply into newly planted habitat and discover most host individuals even as they occurred at very low density. It is possible in the present study that some very early-hatching host individuals had completed larval development altogether before the population was sampled and that, among such individuals, those occurring most deeply within the field indeed most frequently escaped parasitism. Previously [51], however, sampling was begun even earlier in the season, in mid-May when only very recently hatched CLB occurred on the foliage (four-fifths first instar and one-fifth second instar). Even at this very early point in the season, nearly one-third of first instars and three-fourths of second instars had already been discovered and parasitized by *T. julis*, with no significant difference in rate of parasitism among distances varying up to 600 m into a newly planted wheat field [51].

The ichneumonid parasitoid *B. curculionis*, also a relatively small parasitoid (3–4 mm adult length), similarly penetrated as strongly as its host into newly planted alfalfa early in the growing season. Thus, the rate of parasitism varied little with location up to 250 m (the maximum distance studied) into a new field in which alfalfa weevil larvae were abundant and overall parasitism was low (13%), and up to 700 m (again, the maximum distance studied) into another new field in which the weevils were much less abundant and overall parasitism was high (84%). Furthermore, in both cases, the rates of parasitism among early maturing weevil larvae were virtually the same in the newly planted alfalfa as in adjacent previously planted alfalfa. Thus, while both of the pests dispersed rapidly and extensively into newly planted crops, so too did their specialist biocontrol agents, such that neither in wheat or alfalfa fields did the pest escape initially from its parasitoid in colonizing new habitat.

The ability of *T. julis* and *B. curculionis* adults to disperse hundreds of meters into new habitat just as strongly as their hosts in the present study is perhaps to be expected, given the well-documented rapid dispersal across large landscapes of these two biological control agents upon their initial release in North America [46,94]. In addition, these species are host-specific parasitoids of highly dispersive herbivores that exploit frequently disturbed ephemeral habitats. Natural selection can be expected to have instilled strong dispersal ability in these natural enemies just as it has in their hosts [91,95,96,97].

Very high rates of parasitism in newly planted fields were observed in particular for *T. julis* attacking CLB. This might reflect especially strong selection for dispersal ability associated with a habitat templet [98] of annual cropping (small grains) characterized by extreme levels of disturbance (versus perennial cropping of alfalfa). However, other factors seem likely at play as well. Multiple species of hyperparasitoids inflict heavy mortality on *B. curculionis* in western North America, thereby likely limiting its effectiveness against the alfalfa weevil [36,99]. In contrast, no hyperparasitoids have been documented to attack *T. julis* in western North America. In addition, the life cycle timing of *T. julis* results in very high parasitism early in the season (i.e., when the present study was conducted) and substantially lower parasitism as the season progresses [48,50].

## 5. Conclusions

Differences in dispersal rates of insect pests and their natural enemies may compromise biological control in short-lived crops subject to frequent disturbance, if the enemies lag significantly behind the pests in colonizing and establishing populations in newly planted fields. In the present study, however, the two specialist parasitoids, *T. julis* and *B. curculionis*, were strong dispersers that matched their hosts well in moving rapidly over hundreds of meters in the spring into new habitat. Consequently, these natural enemies succeeded in finding and parasitizing the cereal leaf beetle and alfalfa weevil deep into newly planted fields of wheat and alfalfa. It thus appears that neither pest experienced lower risk of parasitism even in the short-term, in dispersing to new habitat patches. 

Two general points should be noted in placing these results in broad perspective. First, the present study was focused on dispersal into newly planted crops by specialist pests and their specialist natural enemies. Different results might well obtain for interactions of generalist pests and generalist natural enemies as they colonize short-lived crops. These species are less tightly linked in their population dynamics, and they likely respond to landscape heterogeneity in more complex fashion, than host- and habitat-specific species such as those studied here [100]. These factors may lead to greater disparity in dispersal characteristics of these generalist species, such that the pests more effectively escape from their enemies in colonizing newly created habitat than do the pests studied here (but see [91]).

It also bears noting that the present study of dispersal is focused specifically on host and parasitoid colonization of newly planted field crops early in the growing season. Dispersal of these species at other times and under other circumstances (e.g., following cutting in alfalfa, or to and from non-crop habitat providing nectar) remains to be explored. Such exploration added to the studies conducted here will enable fuller assessment of the importance of dispersal among habitat patches in influencing both local and regional population dynamics of these host pest-parasitoid interactions.
